# Innate Cell-Mediated Cytotoxic Activity of European Sea Bass Leucocytes Against Nodavirus-Infected Cells: A Functional and RNA-seq Study

**DOI:** 10.1038/s41598-017-15629-6

**Published:** 2017-11-13

**Authors:** Elena Chaves-Pozo, Yulema Valero, Anna Esteve-Codina, Jèssica Gómez-Garrido, Marc Dabad, Tyler Alioto, José Meseguer, M. Ángeles Esteban, Alberto Cuesta

**Affiliations:** 1 Centro Oceanográfico de Murcia, Instituto Español de Oceanografía (IEO), Carretera de la Azohía s/n, Puerto de Mazarrón, 30860 Murcia, Spain; 2 CNAG-CRG, Centre for Genomic Regulation (CRG), Barcelona Institute of Science and Technology (BIST), Baldiri i Reixac 4, 08028 Barcelona, Spain; 3 Universitat Pompeu Fabra (UPF), Barcelona, Spain; 4 Fish Innate Immune System Group, Department of Cell Biology and Histology, Faculty of Biology, Campus Regional de Excelencia Internacional “Campus Mare Nostrum”, University of Murcia, 30100 Murcia, Spain; 5 Present Address: Grupo de Marcadores Inmunológicos, Laboratorio de Genética e Inmunología Molecular, Instituto de Biología, Pontificia Universidad Católica de Valparaíso, Valparaíso, Chile

## Abstract

Nervous necrosis virus (NNV) causes high mortalities in several marine species. We aimed to evaluate the innate cell-mediated cytotoxic (CMC) activity of head-kidney leucocytes (HKLs) isolated from naïve European sea bass (*Dicentrarchus labrax*) and gilthead seabream (*Sparus aurata*), a very susceptible and resistant fish species to NNV, respectively, against fish cell lines infected with NNV. Seabream HKLs showed significantly increased innate CMC activity against NNV-infected cells, compared to those uninfected, while sea bass HKLs failed to do so. Thus, we performed a RNA-seq study to identify genes related to the CMC activity of sea bass leucocytes. Thus, we found that sea bass HKLs incubated with DLB-1 cells alone (CMC_DLB1) or with NNV-infected DLB-1 cells (CMC_DLB1-NNV) showed very similar transcriptomic profiles and the GO analysis revealed that most of the up-regulated genes were related to immunity. Strikingly, when the CMC samples with and without NNV were compared, GO analysis revealed that most of the up-regulated genes in CMC_DLB1-NNV samples were related to metabolism and very few to immunity. This is also in agreement with the functional data. These data point to the escape of CMC activity by NNV infection as an important factor involved in the high susceptibility to nodavirus infections of European sea bass.

## Introduction

In modern aquaculture, a sector in continuous expansion, viruses are the most threatening pathogens since they produce great mortalities and no preventive or therapeutic treatments are available. Among such virus, nervous necrosis virus (NNV; *Nodaviridae* family, *Betanodavirus* genus), or nodavirus, causes fish encephalopathy and retinopathy (VER) altering the structure and functioning of the central nervous system (brain and retina). NNV is a non-enveloped, about 30 nm icosahedric virus with two molecules, RNA1 and RNA2, of single-stranded positive-sense RNA, which are capped but not polyadenylated^1,2^. The RNA-dependent RNA-polymerase (RdRP) is codified by the RNA1 (3.1 kb), which also codifies for the B2 protein (by the subgenomic fragment RNA3) only present in recently infected cells but not in viral particles^2^. The capsid protein (CP) is encoded by the RNA2 (1.4 kb)^3^. To date, NNV is considered the most devastating viral diseases affecting to more than 120 fish species, mainly to larvae and juvenile stages of marine fish species^4,5^. Among them, in the Mediterranean area, European sea bass (*Dicentrarchus labrax*) is highly vulnerable whilst gilthead seabream (*Sparus aurata*), cohabitating in the same areas and/or farms, is a resistant species and a carrier of the infection for most of the NNV strains^6^ though it can suffer some mortalities when infected with certain NNV reassortant strains^7^. Unfortunately, host factors ascribed to this NNV susceptibility are still under debate.

Several mechanisms or pathways of the immune response are greatly involved in the viral defense. In fish, most studies have focused on the IFN pathway^8–10^ including its characterization and regulation though the cell-mediated cytotoxic (CMC) activity has received little attention and merits further characterization since it is the main cellular immune response against virus-infected cells. In teleost fish, different populations of leucocytes are involved in this CMC activity including nonspecific cytotoxic cells (NCCs), natural killer (NK)-like cells and cytotoxic-T lymphocytes (CTL)^11,12^. At functional and cellular levels, they are classified according to innate/adaptive activity and the presence of surface markers. Thus, innate CMC activity is played by NCCs, that express the NCC receptor protein-1 (NCCRP-1) marker, and NK-like cells, which does not^13,14^. The specific CMC response is played by CTLs, which express the T-cell receptor (TCR) and CD8 co-receptor^11,12^. Unfortunately, the implication of fish cytotoxic cells to fight virus has focused very limited attention. The scarce studies have documented increased CMC activity upon *in vitro* or *in vivo* viral infections, as well as the up-regulation of genes related to the CMC activity^15^. In the case of NNV, we have demonstrated that the innate CMC or NCC activity of head-kidney (the main hematopoietic tissue in fish) leucocytes (HKLs) from NNV-infected specimens was increased against xenogeneic tumor cells in both gilthead seabream and European sea bass, but mainly in the last one, and that the gene expression of *nccrp1*, involved in leucocyte recognition of the target cell and binding, was mainly up-regulated in the head-kidney and brain from European sea bass and slightly in the gilthead seabream specimens^16^. These findings suggest that sea bass leucocytes are armed and fight the NNV infection in the brain more efficiently than in seabream, but they still suffered high mortalities. By contrast, most of the other genes studied [chemokines, cytokines, interferon (IFN), Toll-like receptors (TLRs), antimicrobial peptides (AMPs), etc.] were much more up-regulated in the brain of seabream compared to the very low regulation in the sea bass specimens infected with NNV^10,16–22^. Moreover, sea bass genes related to IFN^10,16^ or inflammatory^20^ response were not affected except the *mx* transcription that was very high at 1 day and decreased afterwards, the same pattern than *nccrp1*. By contrast, in seabream brain, there was not significant inflammation and the IFN response increased with the infection time^10,16,20^. Interestingly, while NNV replication was very low and kept steady during infection in the brain of seabream, in sea bass brain its replication was very high at 1 day of infection and highly increased thereafter^16^. Altogether, these data partially explained the high susceptibility to sea bass to NNV in contrast to what happened with seabream. Regarding the specific CMC, infected orange-spotted groupers (*Epinephelus coioides*) increased the *cd8* gene expression as well as the number of CD8^+^ circulating lymphocytes and the specific CMC activity against NNV-infected cells, in a process that was dependent on the MHC I^23^. By contrast, the expression of T cell receptor (*tcr*) and *cd8* genes in European sea bass and Atlantic halibut (*Hippoglossus hippoglossus*) after NNV infection was unaltered, suggesting that this specific CMC activity is not generated in all the fish species^19,24^. Further studies need to be performed to understand the differential immune responses in the brain of susceptible and reservoir fish species and the host-NNV interactions.

Lately, the -omic technologies have been successfully applied to fish studies allowing us to understand and have a deeper knowledge at gene and protein levels of molecular and cellular processes. In order to look for potential candidates in this host-NNV interaction, the European sea bass genome assembly and annotation^25^ has become a very useful resource for the study of several aspects of this fish’s biology. Therefore, with this study, we aimed to improve the European sea bass genome annotation by means of next-generation sequencing (NGS) using RNA-seq analysis from, for the first time in fish, sea bass CMC samples. Thus, several fish cell lines (SAF-1, SSN-1, E-11 and DLB-1) susceptible to NNV, mock- or NNV-infected, were incubated with gilthead seabream or European sea bass freshly isolated head-kidney leucocytes to assess their innate CMC activity *in vitro*. Based on the functional data on sea bass CMC activity against NNV-infected cells we have evaluated the transcriptomic profile in an effort to throw some light in the reasons behind the great susceptibility to nodavirus in this species.

## Results

Fish used in these experiments were reared at the IEO installations which are considered to be NNV-free. Anyway, 10 fish were sampled and assayed for NNV presence by qPCR and immunohistochemistry as well as for antibodies by ELISA as previously^20^ and negativity was confirmed.

### NNV differently replicates in the fish cell lines

**Figure 1:**
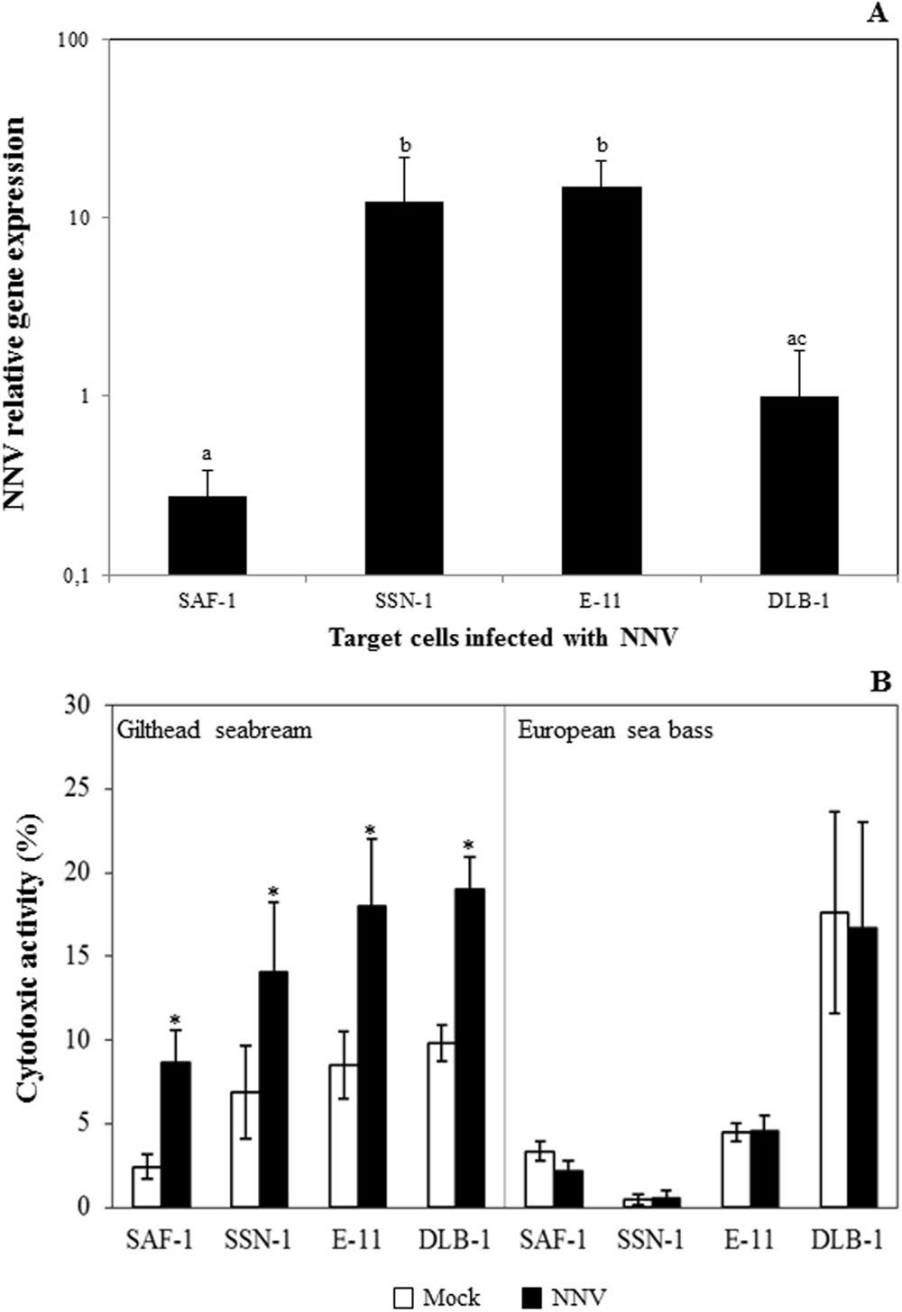
Functional CMC activity. (**A**) The capsid protein (*cp*) gene expression of NNV in the different target cells used in the cytotoxic assays after 24 h infection with 10^6^ TCID_50_ NNV/mL. Data are shown as the mean gene expression relative to the expression of endogenous control *ef1a* gene ± SEM (n = 3). Different letters stand for statistically significant differences (ANOVA; P ≤ 0.05). (**B**) Cytotoxic activity of gilthead seabream or European sea bass isolated head-kidney leucocytes incubated for 4 h with SAF-1, SSN-1, E-11 or DLB-1 cells, mock- (control) or NNV-infected for 24 h with 10^6^ TCID_50_ NNV/mL as determined by the LDH assay. Results are expressed as mean ± SEM (n = 8). Asterisk denotes statistically significant differences (t-Student; P ≤ 0.05) between mock- and NNV-infected groups.

First of all, we checked that the 24 h infection with NNV resulted in efficient infection in all cell lines (Fig. 1A). As expected by their different susceptibility to the virus^26–28^, we found that SAF-1 cell line was the less permissive to the virus and the SSN-1 and E-11 the most, as evidenced by the expression of the NNV *cp* viral gene expression. The DLB-1 cell line, derived from the European sea bass brain^29^, is also susceptible to NNV infection and replication and was used for RNA-seq studies.

### CMC activity of sea bass leucocytes is not primed by NNV infection

The LDH release assay was used to determine the innate CMC activity of gilthead seabream and European sea bass leucocytes (Fig. 1B). This activity of gilthead seabream HKLs was low in gilthead seabream HKLs against SAF-1, SSN-1, E-11 or DLB-1 mock-infected cells, but interestingly it was significantly enhanced against NNV-infected cells, as demonstrated in other fish-virus models^15^. On the other hand, European sea bass HKLs CMC activity against the same targets was similarly detectable but it was not changed by the NNV infection when compared to the mock-infected cells indicating that CMC activity is not primed by NNV infection of target cells.

### Improvement of the sea bass genome annotation

**Figure 2:**
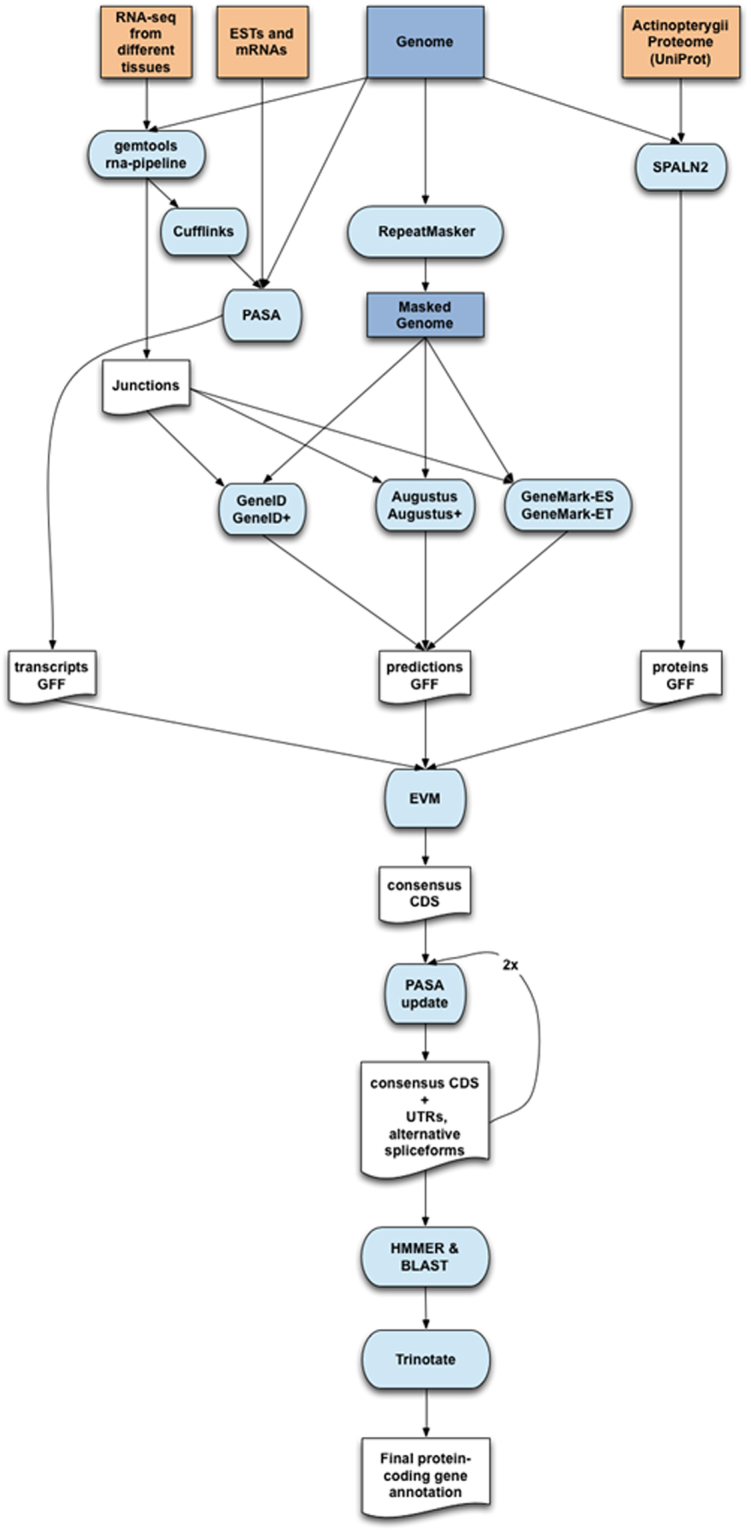
Overview of the annotation pipeline. Input data for annotation are shown at the top of the flow chart. Computational steps are shown in light blue and intermediate data are shown in white.

The RNA-seq analysis resulted in 50–55 million reads per sample comprising a yield of 10–11 Gb. From this we produced a new integrative and high quality genome annotation (Fig. 2) with 25,352 protein coding genes, whose 39,717 transcripts encode 38,069 unique protein products (~1.57 transcripts per gene), whilst the existing genome annotation was made of 26,717 protein-coding genes but only one isoform per gene. In Table 1 we compare some general statistics of both protein-coding annotations. Structural aspects such as exon and intron length are very similar in both cases, which reveal the robustness and high quality of both annotation methods. However, we have annotated less single exon genes, which can occasionally be the result of *ab initio* only gene predictions, without supporting evidence. On the other hand, almost all the genes in the previous annotation contain UTRs in at least one of the ends, which could explain the small increase in number of exons per gene in that annotation. Although we have more annotations with the UTRs missing, the ones that we have annotated tend to be longer.

**Table 1:** Comparison of protein coding gene annotations.

	Previous annotation	New annotation
Number of genes	26,717	25,352
Number of isoforms	26,717	39,792
Exons per gene	9.1	8.9
Multi-exonic genes	0.89	0.94
Median exon length	134	132
Median intron length	367	369
Number of genes with 5′UTR	14,770	10,276
Median length of 5′UTR	191	247
Number of genes with 3′UTR	25,873	11,425
Median length of 3′UTR	387	1,135

**Figure 3:**
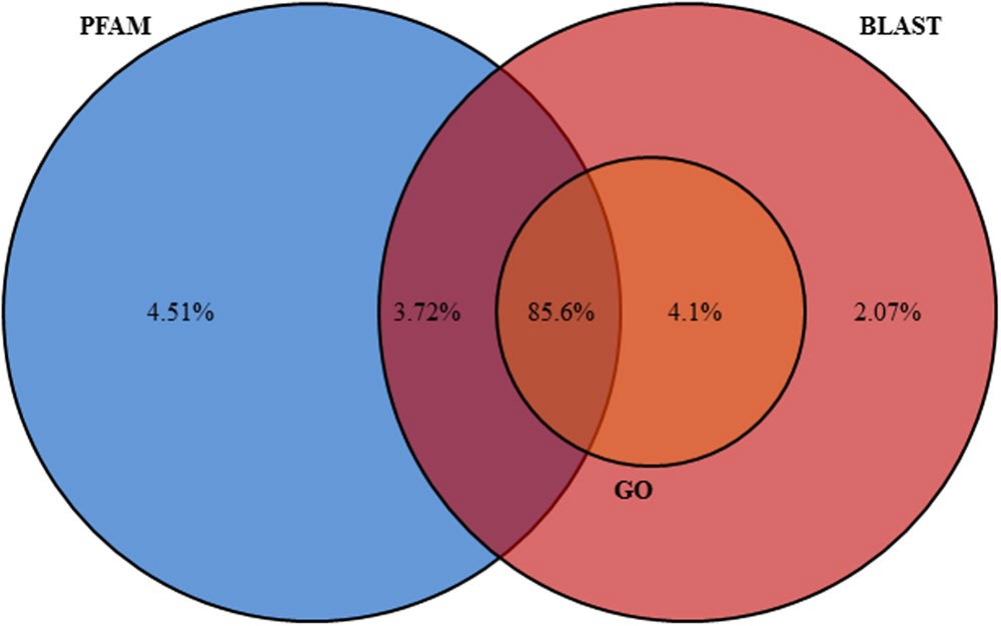
Functional annotation data. Venn diagram showing results of the different functional annotation steps.

After functional annotation 23,947 of the genes were assigned to a functional category (Fig. 3). Gene ontologies could be determined for 21,479 genes (85.7% of the functionally annotated genes) and were further used to extract more relevant and applicable conclusions of the differential expression analysis by gene ontology enrichment.

### Transcriptomic profile in CMC assays reveals up-regulation of immune-related genes

**Figure 4:**
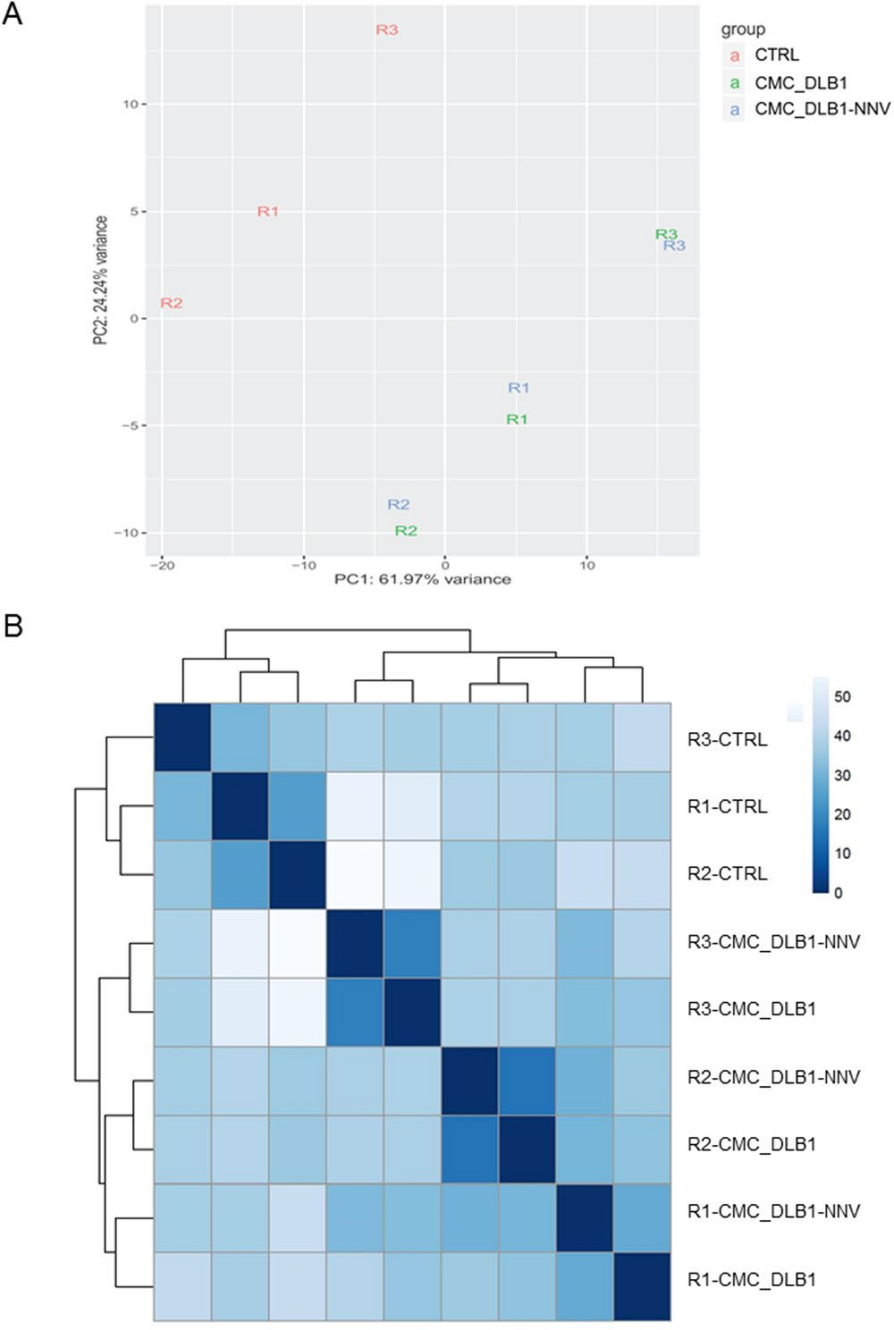
Analysis of the samples similarity. (**A**) Principal component analysis (PCA) with the top most variable genes. (**B**) Heatmap of the samples. Euclidean distances were calculated from the rlog-transformed matrix. From both visualizations, we see that the differences within the same replica fish are smaller than differences between conditions. This shows why it is important to block for the fish replica effect by using the design formula ~ replica+ condition in the differential expression analysis.

**Figure 5:**
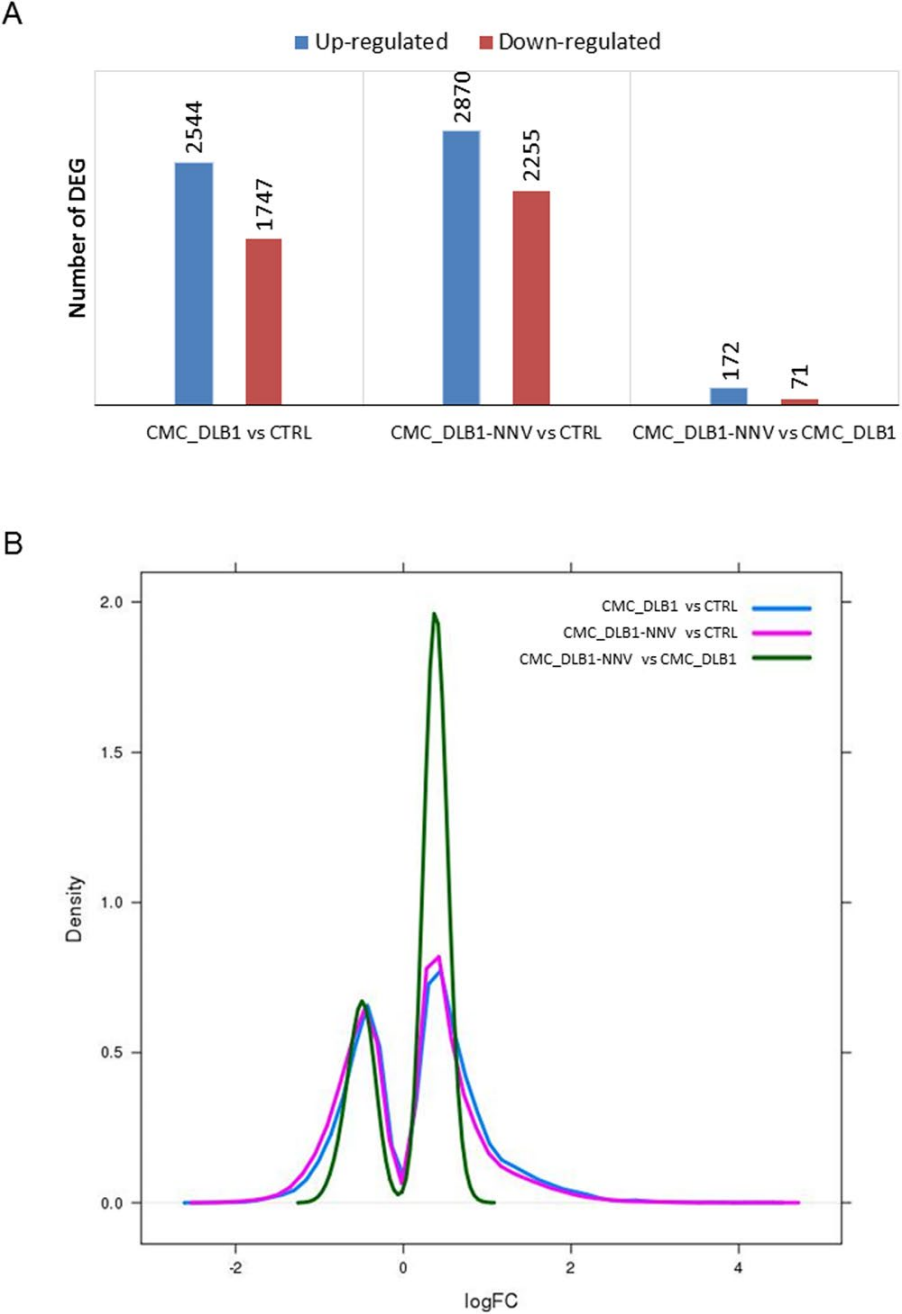
Differentially expressed genes I. (**A**) Bar plots showing the number of up-regulated and down-regulated genes for each comparison. (**B**) Density plots showing the log2FC distribution for each comparison.

Principal component analysis revealed major differences between the European sea bass CTRL and CMC samples but subtle differences between the CMC_DBL1 and CMC_DBL1-NNV conditions (Fig. 4A). Biological replicas coming from 3 different fish (R1, R2 and R3) clustered very close (Fig. 4A,B) suggesting a batch effect. In agreement with this exploratory analysis, more than 4,000 differentially expressed genes (DEG) were detected for each of the CMC assays versus the CTRL (Fig. 5A) but only 242 DEGs with weak effects (Fig. 5A,B) were found between the CMC assays with NNV-infected and mock-infected target cells (Supplementary Table S2).

**Figure 6:**
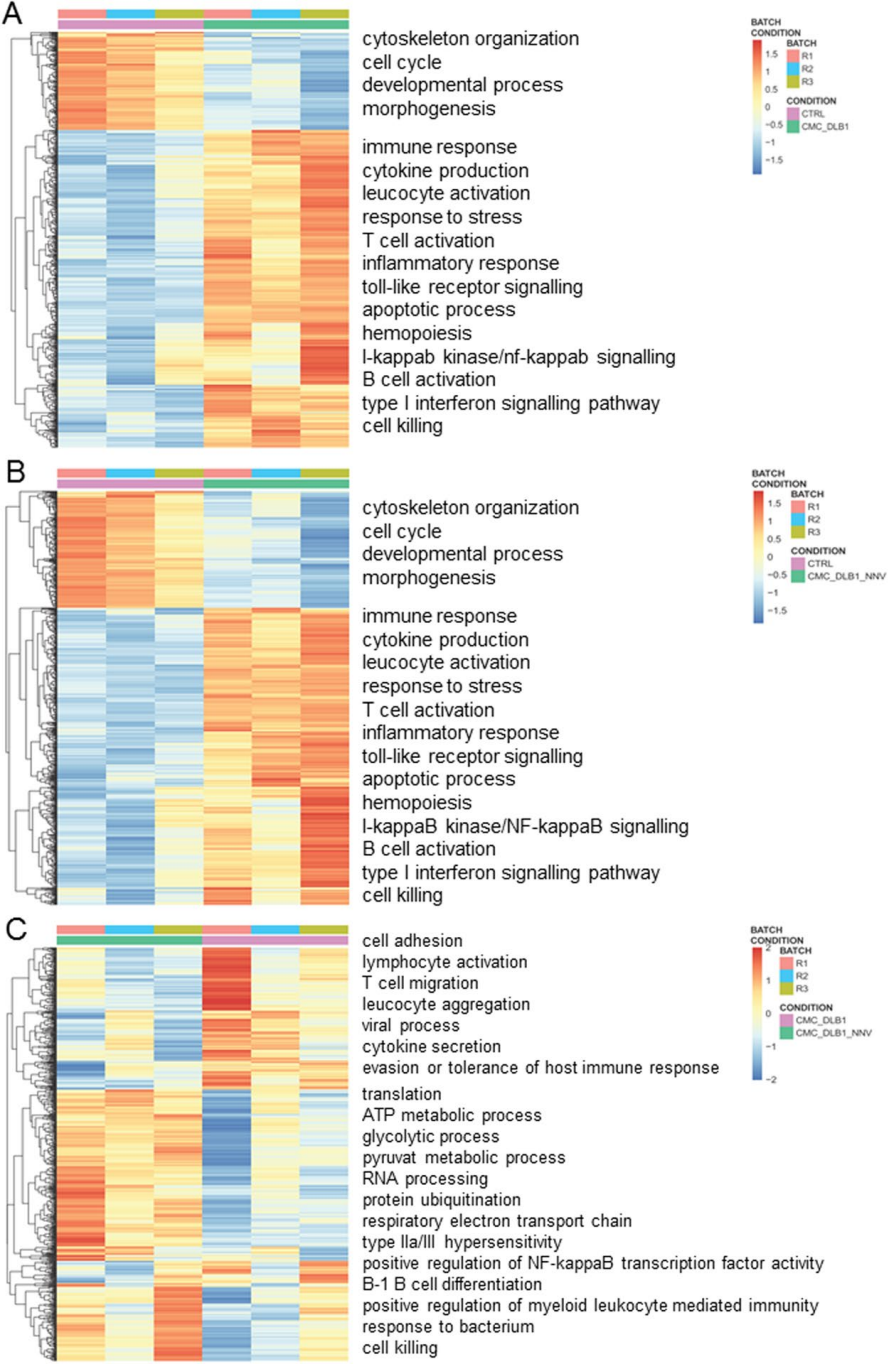
Differentially expressed genes II. (**A**) Heatmap showing scaled expression values of the differentially expressed genes for the CMC_DLB1 *vs* CTRL comparison. (**B**) Heatmap showing scaled expression values of the differentially expressed genes for the CMC_DLB1-NNV *vs* CTRL comparison. (**C**) Heatmap showing scaled expression values of the differentially expressed genes for the CMC_DLB1-NNV *vs* CMC_DLB1 comparison. In all cases, top enriched GO are depicted for up and down-regulated genes.

**Figure 7:**
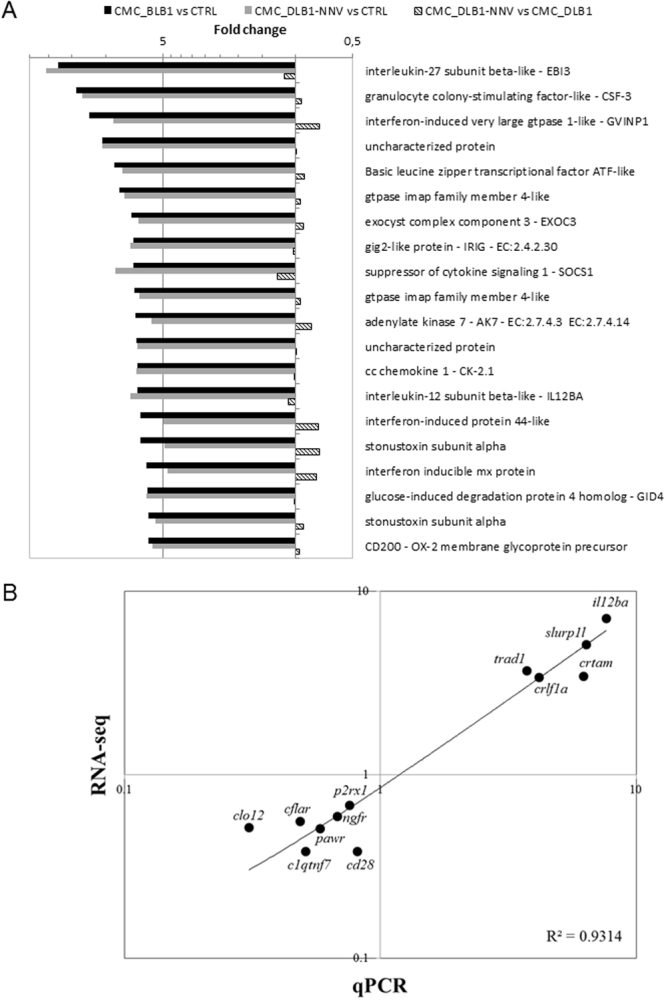
Validation of RNA-seq data. (**A**) Fold change of the 20 top up-regulated genes in the European sea bass CMC_DLB1 samples and their comparison in the other samples. (**B**) Validation of the RNA-seq data by means of the qPCR. Plot represents the mean value for selected genes in each group. Data were fitted by linear regression and adjusting quality determined.

We first evaluated the DEGs in CMC assays respect to the controls (Supplementary Table S2). We found similar number of up- and down-regulated genes (Fig. 5A). The heat maps for both CMC assays compared to the controls were also quite similar and in most of the genes showed similar fold changes (Fig. 6A,B). To gain insights into the biological functions altered in each situation a GO enrichment analysis was performed for the up-regulated and down-regulated genes separately (Fig. 6A,B; Supplementary Table S3). Full GO enrichment list can be in found in Supplementary Table 3. Up-regulated genes in either the CMC assays compared to CTRL were related to immune response, whereas down-regulated genes were involved in cell cycle and development. Among the top up-regulated DEGs we found many genes related to immunity (encoding EBI3, GVINP1, IL12BA, SOCS1, Mx, CK-2.1, CRTAM, SLURP1L, TBX3, etc. proteins) including T-cell biology, IFN-pathway or chemokines (Fig. 7A) which were very similar in either CMC assays against mock- or NNV infected target cells. In addition, some up- and down-regulated genes were selected and its expression also evaluated by qPCR in order to validate the RNA-seq data. Thus, we used 5 up- and 7 down-regulated genes and both RNA-seq and qPCR values were adjusted with an R^2^ = 0.93 demonstrating a good correlation between both RNA-seq and qPCR techniques (Fig. 7B).

### Minor transcriptional effects caused by the NNV infection

**Figure 8:**
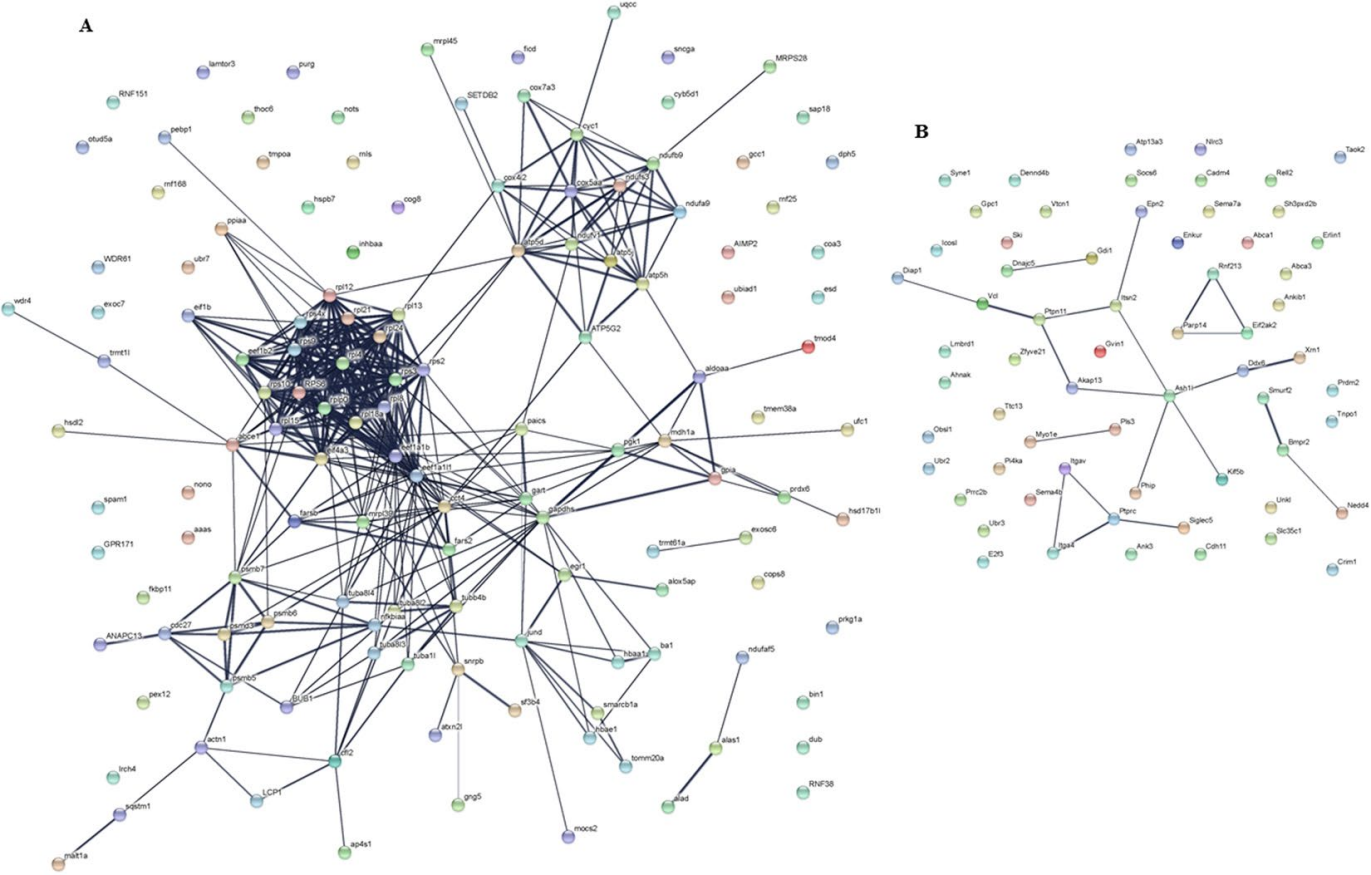
Protein-protein network interactions. (**A**) String analysis for the up-regulated genes in CMC_DLB1-NNV for the CMC_DLB1-NNV *vs* CMC_DLB1 comparison. (**B**) String analysis for the down-regulated genes in CMC_DLB1-NNV for the CMC_DLB1-NNV *vs* CMC_DLB1 comparison.

The European sea bass isolated leucocytes are able to equally kill mock- or NNV-infected target cells as evidenced by the functional and RNA-seq analysis. In the next step, we wanted to ascertain why this CMC activity is not increased by sea bass leucocytes when the target cells are infected by NNV. Thus, we compared the transcriptional profile in CMC-DLB1-NNV *vs* CMC_DLB1 samples. Thus, we found very little number of DEGs (Fig. 5A; Supplementary Table S2). Although the presence of NNV in the CMC assay does not seem to cause major differences in the transcriptome, up-regulated genes in CMC_DBL1-NNV condition were mainly involved in translation, respiratory electron chain and protein ubiquitination and a minority of them were involved in immune-related processes (Fig. 6C, Supplementary Table S3). Protein-protein interaction network analysis showed highly connected clusters of genes, each representing a different biological function (Fig. 8A). Conversely, down-regulated genes in the CMC_DBL1-NNV condition were related to the viral process and transmission and other host immune responses. In that case, the string analysis showed a less connected network (Fig. 8B).

Immune-related differentially expressed genes between the non-infected and infected CMC assays (Table 2) could be potential candidates to explore the inefficient performance of the European sea bass against the NNV-infected cells. Of these, only 2 were not detected as significant in either the CMC assays compared to the CTRL. Others were significantly up-regulated in both the CMC_DBL1 *vs* CTRL and CMC_DBL1-NNV *vs* CTRL but with stronger effects for the second comparison, i.e, the ALOX5AP gene. The direction of the effects and significance for any of the comparisons are shown in Table 2. Noteworthy, any gene had significant opposite directions with respect to CTRL, which reinforces the minimal differences between both CMC assays.

**Table 2:** Descriptions and GO term classification of the differentially expressed genes related to immune response for CMC_DLB1-NNV *vs* CMC_DLB1 comparison.

**Description**	**Gene ontology terms**	**CMC_DBL1 vs CTRL**	**CMC_DBL1-NNV vs CTRL**	**CMC_DBL1-NNV vs CMC_DBL1**
histone h2a-like	response to bacterium, cell killing	not significant	not significant	up
fc receptor-like b-like	type IIa hypersensitivity, type III hypersensitivity, antibody-dependent cellular cytotoxicity, response to bacterium, positive regulation of acute inflammatory response to antigenic stimulus, positive regulation of myeloid leukocyte mediated immunity	not significant	up	up
arachidonate 5-lipoxygenase-activating protein (ALOX5AP)	leukotriene production involved in inflammatory response, arachidonic acid metabolite production involved in inflammatory response	up	up	up
histone partial	response to bacterium, cell killing	not significant	up	up
mucosa associated lymphoid tissue lymphoma translocation gene 1 (MALT1)	B-1 B cell differentiation, positive regulation of NF-kappaB transcription factor activity	up	up	up
neural precursor cell developmentally down-regulated 4 (NEDD4)	cytokine-mediated signaling pathway, immune response, viral process, leukocyte activation, transmission of virus, T cell aggregation, lymphocyte aggregation, dissemination or transmission of symbiont from host, leukocyte cell-cell adhesion	up	not significant	down
integrin alpha-4-like (ITGA4)	leukocyte cell-cell adhesion, immune system process, T cell migration, lymphocyte migration	not significant	down	down
pleckstrin homology domain-containing family a member 1-like (PKHA1)	immune system process, antigen receptor-mediated signaling pathway	up	not significant	down
interferon- double-stranded rna-activated protein kinase-like (PKR)	cytokine production, viral process, immune response, evasion or tolerance by virus of host immune response, regulation of NLRP3 inflammasome complex assembly, regulation of hematopoietic stem cell proliferation, response to interferon-alpha	up	up	down
suppressor of cytokine signaling 6 (SOCS6)	leukocyte cell-cell adhesion, lymphocyte activation, immune system process, T cell aggregation, T cell activation	not significant	down	down
semaphorin-7a-like isoform ×1 (SEMA7A)	immune system process, cytokine production, regulation of macrophage cytokine production	up	not significant	down
bone morphogenetic protein receptor type-2 (BMPR2)	lymphatic endothelial cell differentiation	down	down	down
Poly(ADP-ribose) polymerase family member 14 (PARP14)	T cell aggregation, T cell activation, cytokine production, viral process, regulation of immune response, interleukin-27-mediated signaling pathway, negative regulation of interleukin-6-mediated signaling pathway, regulation of defense response	not significant	not significant	down
receptor-type tyrosine-protein phosphatase c-like (PTPRC)	immune system process, antigen receptor-mediated signaling pathway	not significant	down	down
unconventional myosin-ie (MYO1E)	immune system process	down	down	down
cell adhesion molecule 4-like	immune system process, cytokine production	up	up	down
nucleic acid binding protein	lymphocyte activation, T cell migration, immune system process, T cell aggregation, T cell activation, cytokine production, viral process	up	up	down
protein nlrc3-like	leukocyte cell-cell adhesion, immune system process, T cell migration, lymphocyte migration, T cell activation	up	not significant	down

## Discussion

Previous data about fish CMC activity have shown that cytotoxic activity as well as the expression of relevant-related genes such as perforin or granzymes is increased to a greater extent when fish are infected with virus^15^. However, no studies have attempted to characterize the CMC response against virus in gilthead seabream and European sea bass. Our study, apart from being the first in these fish species, could represent a very good model to understand the differences in such cellular innate immune activity against NNV-infected cells due to the differential resistance of both fish species. In the present study, once confirmed the NNV replication in the DLB-1 cells, we determined the innate cytotoxic activity of gilthead seabream and European sea bass HKLs against them, and compared to the mock-infected or control target cells. Strikingly, this innate CMC activity was increased by seabream HKLs against NNV-infected cells but not in the case of sea bass HKLs. This finding has not been previously reported in fish. Thus, old data available in fish demonstrated that leucocytes from salmonids (Atlantic salmon, *Salmo salar*; rainbow trout, *Oncorhynchus mykiss*) and catfish (*Ictalurus punctatus*) were able to kill virus-infected cells much more efficiently than non-infected cells^30–32^, demonstrating the antiviral activity of fish NCC and NK-like cells, respectively. More recently, in rainbow trout and orange-spotted grouper, CTLs activity against virus was shown^33–35^. Therefore, in the current study we demonstrate for the first time that the European sea bass CMC activity against NNV-infected cells is not primed while this activity did for gilthead seabream. A similar pattern has been also detected in the mammalian natural killer cell function against certain virus-infected cells^36,37^ and represents one of the multiple evasion strategies of viruses, as it can occur with the NNV and sea bass leucocytes. Therefore, and because the cell targets used are exactly the same for both fish species, the reasons determining the differences in the sea bass CMC activity observed must reside in the leucocytes and their functioning. Based on this hypothesis, we have performed a RNA-seq study of the innate CMC activity for the first time in fish in order to 1) know this complex process at molecular level; and 2) understand the molecular basis for the not increased activity against NNV-infected cells in European sea bass leucocytes.

Thus, we first focused on the data about the CMC independently to the targets used. Amongst the most up-regulated genes we found some of them related to immunity with especial implication to T- and NK-cell biology. Therefore, the most up-regulated gene in sea bass CMC assays was interleukin (IL)−27 subunit beta-like (EBI3), that forms part of the heterodimeric IL-27 with the IL-27p28 subunit^38^. This cytokine is produced by antigen presenting cells (APC) and exerts both pro- and anti-inflammatory effects but is readily considered as a potent antitumor molecule. In fact, it increases the NK and CTL proliferation, and their interferon (IFN)γ production, and drives the cells to Th1 responses but at the same time also exerts direct inhibitory effects on tumor cell proliferation, survival, invasiveness, and angiogenic potential^39,40^. This IL-27 subunit was firstly described in the teleost tongue sole (*Cynoglossus semilaevis*)^41^. Its transcription in the head-kidney was increased by bacterial and virus infections and *in vitro* incubation of sole leucocytes with recombinant EBI3 protein resulted in increased respiratory burst and production of IL-1β, IL-8, IFN-stimulated gene 15 (ISG15), chemokines, MyD88 and CD28 as well bactericidal activity. However, in Atlantic salmon, EBI3 was not regulated by pathogen-associated molecular patterns (PAMPs) or recombinant cytokines, whilst the IL-27p28 subunit did^42^. By contrast, we detected the EBI3 transcripts but no for IL-27p28. As important as the IL-27 cytokine, IL-12 bridges innate and adaptive immunity acting as a key regulator of cell-mediated immune responses through the induction of Th1 by promoting IFNγ production, proliferation, and cytolytic activity of NK and CTLs^43^. In sea bass CMC assays we found up-regulated IL-12 beta subunit (IL12BA) and its receptor (IL12RB2). Interestingly, the class I-restricted T cell-associated molecule or cytotoxic and regulatory T cell molecule (CRTAM) has been found to be another marker of cytotoxic cells, including NK, CD8 + CTLs and CD4 + CTLs^44^, which has been up-regulated in sea bass CMC assays. Concomitantly, this CRTAM binds to cell adhesion molecule 1 in the target cells^45^ to drive its cytotoxic function and a neural cell adhesion molecule 1-like has been detected up-regulated, probably in the target DLB-1 target cells used, since they are from brain origin. Another gene up-regulated is the lymphocyte antigen 6-like secreted (SLURP1L) that is secreted and exerts anti-inflammatory and antitumor activities^46^ but this has not been characterized in fish so far. Regarding some of the typical and most known cytotoxic molecules from NK and CTLs, we also found up-regulated genes such as perforin, granzyme A, granzyme B or FasL (tumor necrosis factor ligand superfamily member 6 – FASLG) in the sea bass CMC assays, which are only expressed by sea bass leucocytes and not by the DLB-1 cells. We also found up-regulated the antimicrobial peptide NK-lysin, which is produced by CTLs and NK cells and exerts cytotoxic functions on tumor target cells^47^. This NK-lysin, or granulysin, has been detected in several fish species and related to immune regulation and bactericidal and antiviral activities^48–50^. A very interesting result was the identification of stonustoxin subunit alpha, for the first time in sea bass, as one of the most up-regulated transcripts. This molecule is found in the venom of some fish species and considered an ancient precursor of the perforin, both of them forming pores into cell membranes leading to cell lysis^51^. NCCRP1, the cell marker for fish NCCs and important for target cell recognition and binding^14^, resulted also up-regulated in sea bass CMC assays. Similarly, this mRNA was up-regulated in the brain and head-kidney tissues of NNV-infected sea bass specimens, which also showed increased NCC activity against xenogeneic tumor cells *ex vivo* ^16^. Therefore, the transcriptional profile of sea bass leucocytes under CMC assays reveals for the first time a great up-regulation of many CMC-related genes, including cytotoxic cell markers and cytolytic molecules, which could support the functional cytotoxic activity observed. Additionally, it is well-known that the fish innate CMC response is played by heterogeneous populations of leucocytes (lymphocytes, monocyte-macrophages and/or neutrophil granulocytes)^52,53^ and the establishment of the participation of each leucocyte population to the final activity at both cellular and molecular is uncertain.

Other group of genes important for the immune response were also found up-regulated, including macrophage markers, toll like receptors (TLRs), interleukins or IFN. First, macrophage and/or dendritic cell (DC) markers such as CD83, CD86, MMD2, MRC1 or MARCO, together to major histocompatibility complex (MHC) I and II and related genes, reveals a pivotal role as antigen presenting cells and orchestrating the immunological responses by the expression of many regulatory cytokines and IFNγ. In fact, several cytokines mainly produced by activated macrophages or dendritic cells such as TNFα-related proteins, IL-27, IL-12, IL-6, IL-1β or IL-15 are also up-regulated in sea bass CMC assays. Interestingly, these cytokines exert a remarkable positive regulation of the NK and/or CTL activity in mammals^54^, however no information about their potential role in the fish CMC activity exist at all. Among them, it is worthy to note that IL-15, structurally similar to IL-2, is a pluripotent cytokine that facilitates the generation, proliferation, and function of NK, NK T cells and memory CD8 + T cells^55^. In fish, the scarce literature shows that IL-15 gene is up-regulated after bacterial and viral infections^56^ whilst in rainbow trout recombinant IL-15 induced IFNγ synthesis and recombinant IFNγ induced IL-15 transcription^57^, pointing to the crosstalk between IFNγ and IL-15 in the regulation of cytotoxic cells. However, further studies are needed in fish to identify the interleukin’s role in the CMC response. Regarding TLR transcription, TLRs 1, 3, 5, 9, 8, 13, 18 and 22 were up-regulated in sea bass CMC assays and might have a direct or indirect role on CMC activity. For example, several TLRs are expressed by mammalian NK cells^58^ and CTLs^59^ being TLRs3–9 able to directly enhance the CTL activity and TLR1 indirectly by blocking the Treg functions. Unfortunately, only one paper has observed concomitant up-regulation of TLR9 gene expression and increased CMC activity^60^. Finally, several genes related to the IFN response (GVINP1, IFI30, IF35, IFI44, Mx, IRFs 1, 2, 3, 4, 7 and 10, PKR, CRFB17, etc) were induced in the sea bass CMC assays. Interestingly, these genes were up-regulated to the same extent in CMC samples using DLB-1 cells alone or infected with NNV. This implies that this regulation is not due to the NNV infection and replication and that the levels detected are probably because of their regulation within sea bass leucocytes, where they might play some role in the cytotoxic response, a suggestion that merits further investigations. In fact, it is known that both type I and type II IFNs, namely IFNγ, are positive regulators of the NK and CTLs function in mammals but never evaluated in the fish CMC response^61,62^. All these data points to the importance of cytokines, TLRs and IFNs in the sea bass innate CMC response and the crucial role that might be exerted by macrophages and DC in its regulation, which needs to be undertaken at cellular and molecular levels in fish immunology.

By the action of fish leucocytes, target cells are killed by either apoptosis or necrosis pathways as induced by sea bass innate cytotoxic cells^63^. GO analysis revealed the up-regulation of many transcripts related to the apoptotic process that could fit to the CMC activity regulation, such as caspases 3, 7, 8 and 10, some pro-apoptotic genes such as apoptosis facilitator BCL-2-like protein 14-like, apoptosis-inducing factor mitochondrial-like (AIFM1), apoptosis regulator BCL-X (BCL2L1), apoptosis regulator BAX or apoptosis-enhancing nuclease, TNFα-related proteins and TNF receptors. However, whether these and other apoptosis regulators are mediating target cell death or leucocyte death/survival needs to be established since it is also known that mammalian NK and CTLs^64^ and fish NCCs^65^ also induce their own suicide upon action in order to control the CMC activity. These aspects about apoptosis, or even other killing pathways, should be undertaken and related to the leucocyte-mediated cytotoxic activity in fish.

Finally, after annotation and analysis of the transcripts we found that no significant differences were observed, when compared to controls, among the DEG by target cells alone when compared to the NNV-infected cells indicating that there is not an extra up-regulation in the sea bass HKLs at gene level, as it happened at functional one. In fact, we also searched for transcripts regulated between the two CMC samples, i.e. CMC_DLB1-NNV *vs* CMC_DLB1. Interestingly, very few genes related to immunity were up-regulated and restricted to only H2A, Fc receptor-like b-like, ALOX5AP and MALT1. Thought the functional characterization of fish H2A is limited to antibacterial properties it is able to increase the IL-2 and IL-12 antitumoral activities by mouse NK and CTLs, respectively^66^. Fc receptors are expressed by macrophages, T and B cells and are important to bridge the humoral and cellular immunity being involved in clearance of antigen antibody complex via receptor mediated endocytosis, antibody-dependent cell-mediated cytotoxicity (ADCC), and ligand-triggered transmission of signals across the plasma membrane which results in alteration in secretion, exocytosis and cellular metabolism^67^. However, the cell types expressing Fc receptors and its repertoire is scarcely known in fish. First studies demonstrated that nurse shark IgM mediated opsonisation and innate cytotoxicity^68^ and afterwards the presence of a Fc receptor in catfish NK-like cells was demonstrated to bind IgM and mediate the ADCC response^69^. Thus, it would be worthy to test and probe whether these Fc receptors are mediating ADCC response against NNV-infected cells or not. The paracaspase MALT1 has a central role in the activation of lymphocytes and other immune cells including myeloid cells, mast cells and NK cells leading to their NF-κB-mediated activation, proliferation and survival of the activated immune cells^70^. MALT1, up-regulated in all the sea bass samples, associates with BCL10^70,71^, which is also a DEG in sea bass CMC assays compared to the controls, and activates to NF-κB. Thus, this MALT1-BCL10 could also be partly responsible of the NF-κB-mediated expression of IFNs and cytokines leading to increased innate CMC activity of sea bass leucocytes against tumor or NNV-infected tumor cells. However, other transcripts were down-regulated in CMC assays comparing between NNV-infected and mock-infected target cells. Their relation to CMC response is not well established and more studies should be done in this aspect. For example, SOCS6 suppresses the JAK-STAT-mediated production of cytokines^72^. Thus, we found decreased SOCS6 transcription that would favour the JAK-STAT pathway, cytokine production and finally CMC activity. Following this line, NLR3 is expressed in CD4 + and CD8 + T cells and in NK cells^73^. It reduces the IL-2 mRNA and therefore acts as a negative regulator of the CMC activity. Thus, the down-regulation of these genes in the sea bass leucocyte transcription could partly support the CMC activity data against NNV-infected target cells.

To conclude, this is the first time that the gilthead seabream and European sea bass CMC activity against virus-infected cells has been documented. Interestingly, leucocytes from the susceptible fish species failed to overkill the NNV-infected target cells. This sea bass leucocyte CMC activity was further evaluated by RNA-seq study, being the transcriptomic profile of fish CMC response evaluated for the first time in fish. Up-regulation of many cytotoxic cell-related genes including cell markers, cytokines and cytolytic mediators in sea bass CMC assays against tumor target cells, infected with NNV or not, reveals their importance in the fish CMC response. Unfortunately, very few transcriptomic differences were observed when the CMC against mock- or NNV-infected cells was analysed supporting that the CMC activity is not primed upon infection as also observed at functional levels. Further studies are needed to ascertain why the NNV-infected cells are not more readily killed by sea bass leucocytes and the potential evasion strategies used by NNV to spread in susceptible fish species.

## Material and Methods

### Animals

Adult individuals of the marine teleost gilthead seabream (*Sparus aurata*) and European sea bass (*Dicentrarchus labrax*) (100–150 g mean body weight, bw) were bred at the IEO installations. Fish were transported to the University of Murcia and housed in 450–500 L running seawater (28‰ salinity) aquaria at 24 ± 2 °C with a 12 h light:12 h dark photoperiod during 15 days prior the experiments. Through all the time fish were fed with 1 g per fish once a day using a commercial pellet diet (Skretting).

### Fish cell lines

The established fish cell lines SAF-1^74^, derived from gilthead seabream fin, SSN-1, derived from striped snakehead (*Channa striatus*) whole fry tissue^26^, E-11, a clone derived from the previous SSN-1 cells^27^, and DLB-1, derived from the brain of European sea bass^29^ were used. All of them are susceptible to NNV infections and SAF-1 cells are the only ones that do not contain the snakehead retrovirus (SnRV)^26–28^. Cells were incubated at 25 °C in an atmosphere with 85% relative humidity using L-15 Leibowitz medium (Life Technologies) supplemented with 10% foetal bovine serum (FBS, Life Technologies), except E-11 cells that we used 5% fetal bovine serum (FBS), 2 mM L-glutamine (Life Technologies), streptomycin 100 µg/mL (Life Technologies) an penicillin (100 U/mL, Life Technologies). For SAF-1 and DLB-1 cells, the subculture was done according to standard trypsinization methods (trypsin 0.25%/EDTA 0.02%, Life Technologies) and cells were centrifuged at 400 g for 10 min. For SSN-1 and E-11 cultures, cells were detached by gentle shaking and pipetting. In all cases, cells were counted and viability higher than 95% as determined by the trypan blue staining.

### Nodavirus production

Nodavirus (strain It/411/96, genotype RGNNV) were produced using SSN-1 as host cells at 25 °C until the cytopathic effect was extensive. Supernatant was obtained after centrifugation and viral titer determined in 96-well plates before used in the experiments^75^.

### Leucocyte isolation

For isolation of head-kidney leucocytes (HKLs), gilthead seabream and European sea bass specimens were bleed and after dissection the head-kidney was cut into small fragments and transferred to L-15 culture medium containing 5% FBS, L-glutamine, penicillin and streptomycin. Tissue fragments were pressed to be disorganized and passed by 100 µm nylon mesh to obtain cellular suspensions. Cells were washed twice, counted and adjusted to 10^7^ cells/mL. Trypan blue was used to determine the cell viability.

### CMC assays

SAF-1, SSN-1, E-11 or DLB-1 cells in log growth-phase were detached and counted as above. Cells were then seed in 96-well bottomed-flat plates (Nunc) at a density of 15,000 cells/well without (mock) or with 10^6^ TCID_50_ NNV/mL (NNV-infected) and used as targets. After 24 h of incubation at 25 °C, infection was confirmed by PCR (see below), wells were washed with culture medium and 100 µl of isolated HKLs (effectors) were added at an approximate ratio of 50 HKLs per target cell. Samples were then centrifuged at 400 g for 1 min to favour cell contact and incubated for 4 h at 25 °C. After incubation, samples were processed for determination of the CMC activity. Samples with targets alone or HKLs alone were also used as controls.

CMC activity was determined by the lactate dehydrogenase (LDH) release^76^ using the Cytotox 96^®^ non-radioactive cytotoxicity assay (Promega) according to the manufacturer’s instructions. For this, after incubation, CMC samples were centrifuged at 400 g for 5 min and supernatants collected to assess the LDH activity. The cytotoxic activity was calculated by the following formula:

$$\begin{array}{rcl}{\rm{C}}{\rm{M}}{\rm{C}}\,{\rm{a}}{\rm{c}}{\rm{t}}{\rm{i}}{\rm{v}}{\rm{i}}{\rm{t}}{\rm{y}}\,( \% ) & = & 100\times ({\rm{E}}{\rm{x}}{\rm{p}}{\rm{e}}{\rm{r}}{\rm{i}}{\rm{m}}{\rm{e}}{\rm{n}}{\rm{t}}{\rm{a}}{\rm{l}}\,\mbox{--}\,{\rm{E}}{\rm{f}}{\rm{f}}{\rm{e}}{\rm{c}}{\rm{t}}{\rm{o}}{\rm{r}}\,{\rm{S}}{\rm{p}}{\rm{o}}{\rm{n}}{\rm{t}}{\rm{a}}{\rm{n}}{\rm{e}}{\rm{o}}{\rm{u}}{\rm{s}}\\  &  & \mbox{--}{\rm{T}}{\rm{a}}{\rm{r}}{\rm{g}}{\rm{e}}{\rm{t}}\,{\rm{S}}{\rm{p}}{\rm{o}}{\rm{n}}{\rm{t}}{\rm{a}}{\rm{n}}{\rm{e}}{\rm{o}}{\rm{u}}{\rm{s}})/({\rm{T}}{\rm{a}}{\rm{r}}{\rm{g}}{\rm{e}}{\rm{t}}\,{\rm{M}}{\rm{a}}{\rm{x}}{\rm{i}}{\rm{m}}{\rm{u}}{\rm{m}}\\  &  & \mbox{--}{\rm{T}}{\rm{a}}{\rm{r}}{\rm{g}}{\rm{e}}{\rm{t}}\,{\rm{S}}{\rm{p}}{\rm{o}}{\rm{n}}{\rm{t}}{\rm{a}}{\rm{n}}{\rm{e}}{\rm{o}}{\rm{u}}{\rm{s}})\end{array}$$

### RNA isolation

Total RNA was isolated from samples for real-time PCR gene expression and RNA-seq studies. Thus, we used cytotoxic samples consisting on sea bass HKLs (n = 3 independent and separate) incubated for 4 h with DLB-1 (CMC_DLB1) or NNV-infected DLB-1 (CMC_DLB1-NNV) cells as targets. Controls (CTRL) consisted on CMC samples incubated for 0 h. After incubation, plates were centrifuged (450 g, 5 min, 4 °C), the cells recovered in TRIzol Reagent (Life Technologies) and total RNA isolated by the PureLink^®^ RNA Mini Kit (Life Technologies) with on-column DNase treatment according to the manufacturer’s instructions. The concentration and the quality of RNA were analyzed using a Nanodrop ND1000 (Nanodrop Technologies) and Agilent 2100 Bioanalyzer (Agilent Technologies).

### Stranded mRNA library preparation and sequencing

The libraries from the total RNA were prepared using the TruSeq^®^Stranded mRNA LT Sample Prep Kit (Illumina Inc.) according to manufacturer’s protocol. Briefly, 0.5 µg of total RNA was used for poly-A based mRNA enrichment with oligo-dT magnetic beads. The mRNA was fragmented (resulting RNA fragment size was 80–250 nt, with the major peak at 130 nt). The second strand cDNA synthesis was performed in the presence of dUTP instead of dTTP, to achieve the strand specificity. The blunt-ended double stranded cDNA was 3′-adenylated and Illumina indexed adapters were ligated. The ligation product was enriched with 15 PCR cycles and the final library was validated on an Agilent 2100 Bioanalyzer with the DNA 7500 assay.

Each library was sequenced using TruSeq SBS Kit v3-HS, in paired end mode with the read length 2 × 76 bp. We generated on average 53 million paired-end reads for each sample in a fraction of a sequencing lane on HiSeq. 2000 (Illumina) following the manufacturer’s protocol. Images analysis, base calling and quality scoring of the run were processed using the manufacturer’s software Real Time Analysis (RTA 1.13.48) and followed by generation of FASTQ sequence files by CASAVA 1.8.

### European sea bass genome re-annotation

We performed a new protein-coding gene annotation of the European sea bass genome. For this purpose, we obtained consensus gene models by combining transcript alignments, protein alignments, *ab initio* gene predictions and the current sea bass annotation^25^. A flowchart of the following annotation process can be found in Fig. 2.

RNA-seq data were aligned to the *Dicentrarchus labrax* assembly with GEM v1.7^77^ and transcript models were subsequently generated with Cufflinks^78^. Moreover, sea bass ESTs and mRNAs deposited in public databases were mapped to the genome with gmap (v2014–12–23) and the complete *Actinopterygii* proteome was also mapped using SPALN v2. *Ab initio* gene predictions were performed on the repeat-masked assembly with three different gene prediction programs: GeneID^79^, Augustus^80^ and GeneMark^81^. For GeneID we used the parameter file specific for the *Tetraodon* genus that has been previously used in the past to accurately generate gene predictions in other fish genomes^82^. Augustus v3.0.2 was run with the *Homo* sapiens pre-existing parameter file and GeneMark-ES v2.3e gene predictions were obtained using its self-training mode. Each of the gene predictors was run in two different modes: with and without incorporating intron evidence extracted from the RNAseq data.

All the data detailed above was combined with the current sea bass annotation^25^ to produce a set of consensus CDS models, which were then updated to include UTR sequences and annotate alternative splice forms. In addition, functional annotations were performed on the annotated genes with Trinotate (trinotate.github.io).

### RNA-seq gene quantification

RNA-seq reads were re-aligned with the GEMtools RNA-seq pipeline v1.7 (http://gemtools.github.io), which is based on the GEM mapper^77^, using the improved version of the sea bass annotation (described above). The pipeline aligns the reads in a sample in three phases, mapping against the sea bass reference genome, against a reference transcriptome and against a de novo-transcriptome, which is generated from the input data to detect new junction sites. After mapping, all alignments were filtered to increase the number of uniquely mapped reads. The filter criteria contained a minimum intron length of 20 bp, a maximum exon overlap of 5 bp and a filter step against a reference annotation checking for consistent pairs and junctions were both sites align to the same annotated gene. Mapping statistics and gene expression quantification were calculated using the GEMtools ‘gtfcount’ tool.

### RNA-seq differential expression analysis

Differentially expressed genes (DEGs) were determined with the DESeq2 R package^83^ correcting the replica effect (design ~ replica + condition). Genes with a false discovery rate (FDR) < 5% were considered significant. A gene ontology enrichment analysis was performed with the differentially expressed genes using ‘GOstats’^84^. ‘ggplot2’ R package was used to plot principal component analysis. Heatmaps were done with the ‘pheatmap’ R package. Protein-protein interaction networks were built up with the string database^85^.

### Gene expression by real-time PCR (qPCR)

We evaluated the viral replication in fish cell lines by studying the expression of the NNV *cp* gene and validated the RNA-seq data by the expression of selected genes in CMC assays using real-time PCR (qPCR) and the 2^−ΔCT^ method^86^. For this, total RNA was treated with DNAse I followed by cDNA synthesis using the ThermoScript^TM^ RNAse H- Reverse Transcriptase (Invitrogen) and random hexamers (Invitrogen), ending with a RNAse H (Invitrogen) treatment.

Real-time PCR was carried out with SYBR Green PCR Core Reagents (Applied Biosystems) in an ABI PRISM 7500 instrument (Applied Biosystems). PCR reaction mixtures were incubated for 10 min at 95 °C, 40 cycles of 15 s at 95 °C, 1 min at 60 °C, and a final cycle of 15 s at 95 °C, 1 min 60 °C and 15 s at 95 °C as dissociation curve. To normalize the mRNA content the transcription of the hose-keeping elongation factor 1-alpha (*ef1a*) was determined and expressed as 2^−ΔCt^, where ΔCt is determined by subtracting the *ef1a* Ct value from the target Ct. Gene names follow the accepted nomenclature for zebrafish (https://wiki.zfin.org). The primers used are shown in Supplementary Table S1. Specificity of each primer pair was evaluated using positive and negative samples as well as the sequencing of amplicons. All amplifications were performed in duplicate and repeated once to confirm the results. Samples without cDNA were run in parallel to check potential contaminations.

### Statistical analysis

Figures are presented as mean ± SEM (n = 3) of the data. Statistical differences between groups were analysed by either t-Student or one-way analysis of variance (ANOVA; P ≤ 0.05).

### Ethical statement

The use of animals was approved by the Bioethical Committees of the IEO and the University of Murcia (Permit Number: A13150104). All the *in vitro* assays followed the general guidelines for Good Practice Laboratory principles according to the Organisation for Economic Co-operation and Development (OECD).

### Data availability

The annotation produced in this study can be downloaded from http://denovo.cnag.cat/genomes/seabass/ where we provide also a JBrowse with tracks for all the RNAseq and data used to annotate the genome. The data discussed in this publication have been deposited in NCBI’s Gene Expression Omnibus^87^ and are accessible through GEO Series accession number GSE101662 (https://www.ncbi.nlm.nih.gov/geo/query/acc.cgi?acc=GSE101662).

## Electronic supplementary material

Supplementary Table S1

Supplementary Table S2

Supplementary Table S3
