# Supplementary material for: Innate Cell-Mediated Cytotoxic Activity of European Sea Bass Leucocytes Against Nodavirus-Infected Cells: A Functional and RNA-seq Study
Source: Sci Rep. 2017 Nov 13;7:15396. doi: 10.1038/s41598-017-15629-6 (PMC5684396; doi:10.1038/s41598-017-15629-6)
Supplement: Supplementary file 1 — Supplementary Table S1 [file 41598_2017_15629_MOESM1_ESM.doc]

**INNATE CELL-MEDIATED CYTOTOXIC ACTIVITY OF EUROPEAN SEA BASS LEUCOCYTES AGAINST NODAVIRUS-INFECTED CELLS. A FUNCTIONAL AND RNA-seq STUDY**

**Elena Chaves-Pozo^1^, Yulema Valero^1^, Anna Esteve-Codina^23^, Jèssica Gómez-Garrido^23^, Marc Dabad^23^, Tyler Alioto^23^,· José Meseguer^4^, M. Ángeles Esteban^4^ and Alberto Cuesta^4*^**

**Supplementary Table S1.** Primers used for real-time PCR analysis.

| **Gene name** | **Abbreviation** | **Acc. number** | **Sequence (5’-3’)** |
| --- | --- | --- | --- |
| NNV coat protein | *cp* | D38636 | CAACTGACAACGATCACACCTTC  CAATCGAACACTCCAGCGACA |
| Lymphocyte antigen 6d | *slurp1l* | DLAgn_00102470 | AGCCAGAGTGAAGCCTTGAA  ATGATGACGGTGACACAGGA |
| XIAP-associated factor 1-like - TRAD1 | *trad1* | DLAgn_00028910 | TACCAGCAAACAACCAGCAA  GGGAACACAGTCCAGCTCAT |
| PRKC apoptosis wt1 regulator – PAWR | *pawr* | DLAgn_00209350 | TCAGCATACCATCCAACGAG  GGCCTGAACTAGCTCCTCCT |
| Interleukin-12 subunit beta-like - IL12BA | *il12ba* | DLAgn_00044590 | CACTGCACCTGGACAAGAAA  CGCTGATCCATTAGCACTCA |
| Complement c1q tumor necrosis factor-related protein 7-like - C1QTNF7 | *c1qtnf7* | DLAgn_00152770 | AACCCAGAAGACGAGGTGTG  GGAAGCCAGAGAACAAGCTG |
| Pou class transcription factor 2-like - CL012 | *clo12* | DLAgn_00097250 | CCAACCCACACAGCTCAAC  TACTGCATGCTGTCCTCCTG |
| Cytokine receptor-like factor 1-like - CRLF1A | *crlf1a* | DLAgn_00024100 | TGATGCAGAAATCCCACAAA  GGCTTGGTGAAGTCATGGTT |
| Casp8 and FADD-like apoptosis regulator - CFLAR | *cflar* | DLAgn_00049890 | GACAGATGGGTGCGATAGGT  TAAATGGAGCGATGGTTTCC |
| Tumor necrosis factor receptor superfamily member 16-like - NGFR | *ngfr* | DLAgn_00061300 | TCCCACAGCATACAGGACAA  GAGGCGCTCTACCTCTTCCT |
| T-cell-specific surface glycoprotein CD28 precursor - CD28 | *cd28* | DLAgn_00139520 | TTGCACCCTGAACCCTTAAC  ACTTCTCCCGTCATGTTTGG |
| Cytotoxic and regulatory t-cell molecule - CRTAM | *crtam* | DLAgn_00038630 | CCCACTGGATTTCCTCTGAA  ATTCCTGCTGCTGGTTGAGT |
| P2x purinoceptor 1-like - P2RX1 | *p2rx1* | DLAgn_00039660 | TCGACTGGACATGCAACCTA  GTAGCCCACTGAAGCTCTGG |
| Elongation factor 1 alpha | *ef1a* | FM019753 | CGTTGGCTTCAACATCAAGA  GAAGTTGTCTGCTCCCTTGG |

**Supplementary Table S2.** List of differentially expressed genes (DEGs) in the European sea bass CMC assays. Data represent the mean (n=3). FC, fold change in transcription; FDR, false discovery rate. FDR (or adjusted P value) ≤ 0.05 were considered significant. Not significant FDR values were denoted in red colour. Transcripts undetected in DLB-1 or NNV-infected DLB-1 cells by RNA-seq analyses (data not shown) were also denoted in the last column.

**Supplementary Table S3.** GO enrichment analysis of DEGs in the European sea bass CMC assays.
